# Cytotoxicity Enhancement of α-Mangostin with Folate-Conjugated Chitosan Nanoparticles in MCF-7 Breast Cancer Cells

**DOI:** 10.3390/molecules28227585

**Published:** 2023-11-14

**Authors:** Yedi Herdiana, Nasrul Wathoni, Shaharum Shamsuddin, Muchtaridi Muchtaridi

**Affiliations:** 1 Department of Pharmaceutics and Pharmaceutical Technology, Faculty of Pharmacy, Universitas Padjadjaran, Sumedang 45363, Indonesia; nasrul@unpad.ac.id; 2 School of Health Sciences, Universiti Sains Malaysia, Kubang Kerian 16150, Malaysia; shaharum1@usm.my; 3 Nanobiotech Research Initiative, Institute for Research in Molecular Medicine (INFORMM), Universiti Sains Malaysia, Penang 11800, Malaysia; 4 USM-RIKEN Interdisciplinary Collaboration on Advanced Sciences (URICAS), Universiti Sains Malaysia, Penang 11800, Malaysia; 5 Department of Pharmaceutical Analysis and Medicinal Chemistry, Faculty of Pharmacy, Universitas Padjadjaran, Sumedang 45363, Indonesia; muchtaridi@unpad.ac.id

**Keywords:** chitosan, conjugates, drug release, active targeting, nanocarrier

## Abstract

α-mangostin (AM) is a promising natural anticancer agent that can be used in cancer research. However, its effectiveness can be limited by poor solubility and bioavailability. To address this issue, chitosan-based nanoparticles (CSNPs) have been investigated as a potential delivery system to enhance the cytotoxicity to cancer cells and improve selectivity against normal cells. In this study, we developed folate-conjugated chitosan nanoparticles (F-CS-NPs) using a carbodiimide-based conjugation method to attach folate to chitosan (CS), which have different molecular weights. The NPs were crosslinked using tripolyphosphate (TPP) via ionic gelation. To characterize the F-CS-NPs, we utilized various analytical techniques, including transmission electron microscopy (TEM) to evaluate the particle size and morphology, Fourier-transform infrared spectroscopy (FTIR) to confirm the presence of functional groups, and ultraviolet-visible spectroscopy (UV-Vis) to measure the absorption spectrum and confirm the presence of folate. The particle size of AM-F-CS-NPs ranged from 180 nm to 250 nm, with many having favorable charges ranging from +40.33 ± 3.4 to 10.69 ± 1.3 mV. All NPs exhibited the same spherical morphology. The use of F-CS-NPs increased drug release, followed by a sustained release pattern. We evaluated the cytotoxicity of AM, AM-F-CS-HMW, and AM-F-CS-LMW NPs against MCF-7 cells and found IC_50_ values of 8.47 ± 0.49, 5.3 ± 0.01, and 4.70 ± 0.11 µg/mL, respectively. These results confirm the improved cytotoxicity of AM in MCF-7 cells when delivered via F-CS-NPs. Overall, our in vitro study demonstrated that the properties of F-CS-NPs greatly influence the cytotoxicity of AM in MCF-7 breast cancer cells (significantly different (*p* < 0.05)). The use of F-CS-NPs as a drug-delivery system for AM may have the potential to develop novel therapies for breast cancer.

## 1. Introduction

Breast cancer is a significant cause of mortality among women worldwide [1,2], and current treatment methods, such as chemotherapy, surgery, radiation, and hormonal therapy, have limitations, including lack of effectiveness, damage to healthy organs, and development of drug resistance in cancer cells [3,4]. The tumor microenvironment is a critical factor in cancer therapy. It allows for more significant drug accumulation at the tumor site and targeted and regulated drug release, while maintaining selectivity to normal cells [5]. Mangosteen (*Garcinia mangostana Linn*.) is well known as the queen of fruits, and it grows in the Southeast Asian region. α-mangostin (AM), the main xanthone derivative contained in mangosteen pericarp, has many pharmacological activities, such as antioxidant, antiproliferation, anti-inflammatory, and anticancer [6,7]. AM has demonstrated potential therapeutic benefits for many types of cancer cells, with antitumor effects inhibiting key phases of cancer development [8,9,10,11], increasing cell cycle arrest and death in breast cancer cells [10,11], and growing selectivity [12]. However, AM’s hydrophobic nature, poor water solubility, limited bioavailability [6,13,14,15], and accumulation in target organs pose significant challenges [16,17]. Folate-conjugated chitosan nanoparticles (F-CS-NPs) have been developed to enhance AM cytotoxicity and to selectively target MCF-7 breast cancer cells. Using F-CS-NPs for targeted drug delivery may help to overcome the limitations of conventional treatment methods and increase the effectiveness of AM in breast cancer therapy.

Nanomicelle formation and polymeric nanoparticle encapsulation have been proven to enhance the solubility of AM and facilitate its targeted delivery to specific organs [18]. Various studies, conducted both in vitro and in vivo, have demonstrated the enhanced cytotoxicity, antiproliferative activity, and induction of apoptosis when AM is delivered through these nanoparticle systems, such as transferrin-conjugated lipid–polymer hybrid NPs [19], AM-encapsulated poly(lactic-co-glycolic) acid (PLGA) NPs [20], AM-CS/sodium alginate NPs [21], AM-CS/carrageenan NPs [22] and hyaluronic acid-coated CS-NPs [23]. Additionally, NPs constructed with crosslinked CS and glyoxal have demonstrated controlled-release properties, making them a promising drug-delivery system for hydrophobic compounds such as AM [24].

Modified chitosan nanoparticles (CSNPs) have emerged as a promising drug-delivery system for cancer therapy, addressing critical challenges in bioavailability, stability, cellular uptake, protein adsorption, and drug distribution [25,26,27]. Recent studies have demonstrated that depolymerized CS can increase the cytotoxicity of AM in MCF-7 cells. The properties of CSNPs are influenced by the inherent properties of the CS polymer [28,29]. CS’s unique chemical and physical characteristics determine the degree of crystallinity, surface smoothness, porosity, surface charge, size, and shape. The increased deacetylation degree of CS enhances its hydrophilicity, making it more suitable for formulating drug carriers. As a result, these properties directly affect the drug-loading capacity, release behavior, biocompatibility, targeted delivery, cellular uptake, and overall therapeutic efficacy of CSNPs [30,31].

Folate conjugation is vital in actively targeting cancer cells, as many tumors overexpress folate receptors [32]. This targeted approach enhances drug uptake by cancer cells and reduces damage to healthy tissues, minimizing the side effects associated with conventional chemotherapy [2,4,33]. F-CS-NPs, obtained by attaching folate conjugates to CS using a carbodiimide reaction, have demonstrated increased cytotoxicity against breast cancer cells, highlighting their potential for breast cancer therapy [34]. CS-based drug carriers show great potential in cancer treatment when modified and targeted with folate.

**Scheme 1:**
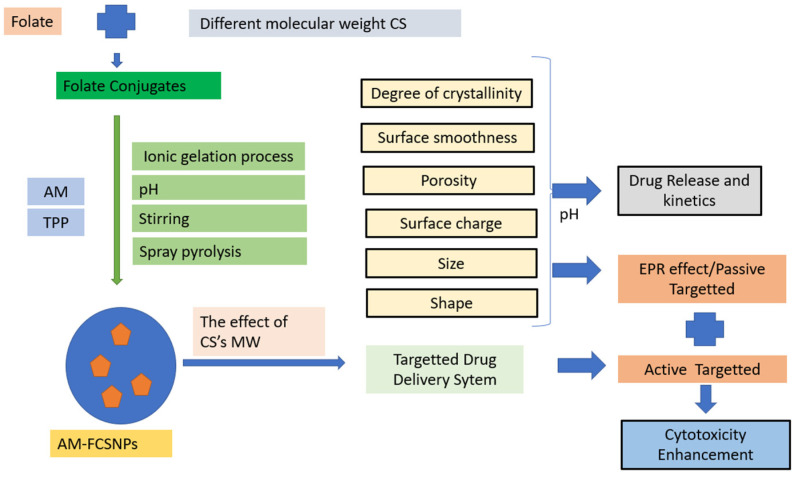
The idea of AM-F-CS NPs as an active, targeted drug.

This research paper aims to further this study by functionalizing CSNPs with folate for targeted delivery of AM using CS, which have different molecular weights. Adding folate will provide a new vector for cancer cells, serving as an active target for drug delivery and enhancing the cytotoxicity of AM in a multimodal manner (Scheme 1). However, further studies are needed to investigate the potential enhancement of the cytotoxicity of AM using F-CS-NPs, based on release studies. This work represents an essential step toward developing nanosystems for treating breast cancer by combining CS, derivatized CS, and natural anticancer drugs.

## 2. Results and Discussion

### 2.1. Characterization of F-CS

The interaction between CS and folate was investigated using FTIR spectroscopy. The measurements were obtained at 4000–400 cm^−1^ under vacuum pressure of 60 kN within 15 min.

**Figure 1:**
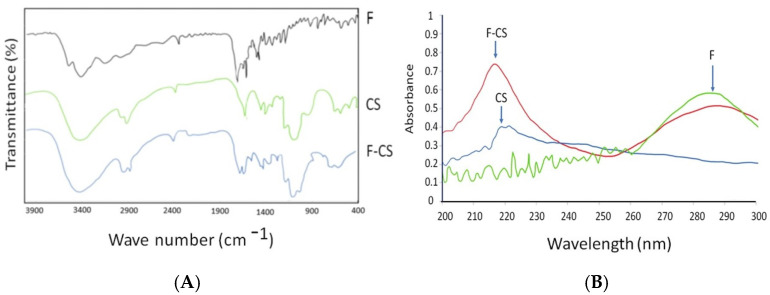
(**A**) IR spectral of F-CS; (**B**) UV spectral absorption of F-CS.

F-CS, as depicted in Figure 1, demonstrates comparable IR spectra to CS due to their structural similarities. CS and F-CS have similar chemical compositions and functional groups, contributing to the overlapping peaks observed in their infrared spectra. Specifically, they exhibit peaks within the 3300- to 3500-cm^−1^ range, corresponding to the stretching vibrations of hydroxyl (OH) groups in both CS and F-CS. Additionally, both materials show peaks at approximately 1642 cm^−1^, representing the stretching vibrations of N-H bonds, further highlighting their shared characteristics. These similarities in the IR spectra suggest that F-CS retains the essential features of CS while undergoing specific modifications or treatments [35]. The presence of an amide bond in F-CS can be identified by the C=O stretching band, which typically appears in the 1650 and 750 cm^−1^ ranges. This band is usually strong and sharp, characteristic of all amide compounds. Therefore, the appearance of the characteristic C=O stretching band in the IR spectra of F-CS confirms the presence of an amide bond between CS and folate. While CS and F-CS should have similar IR spectra, modifying CS with folate may result in a slight shift or change in the intensity of specific peaks, particularly in the regions where the amide bond is present (1650 and 750 cm^−1^). The observed shift in peak intensity can confirm the successful conjugation of folate in CS, ultimately leading to the formation of F-CS.

Peak absorption spectroscopy is a valuable technique for identifying specific substances in a sample and quantifying their concentrations. The peak of the absorption spectrum corresponds to the wavelength at which the substance absorbs light most strongly. One can determine the presence of specific compounds by comparing the absorption spectrum of an unknown sample to known absorption spectra of various substances. In this case, peak absorption at 282 nm could indicate the presence of substances such as amino acids, which typically exhibit a peak absorption at around 280 nm due to aromatic amino acids that absorb light in that region. In the case of F-CS, two absorption bands appeared in the spectral ranges of 200–300 and 300–400 nm, which were assigned to the π-π* and n-π* transitions, respectively (Table 1) [36]. Further analysis using UV-Vis and FTIR spectroscopy confirmed the attachment of folate to CS, as indicated by the characteristic absorption bands of the amide bond in the IR spectra [23]. However, F-CS yield may be reduced due to the sequential processes required in its manufacture, such as dialysis purification. During dialysis, unbound folate and soluble EDC hydrochloride can diffuse outside the dialysis membrane and be removed from the F-CS solution.

### 2.2. Preparation of AM-F-CS-NPs

Folate-functionalized AM-F-CS-NPs with uniform shapes were successfully synthesized using the ionic gelation process. The SEM and TEM data showed that the NPs had a homogenous sub-200 nm spherical shape. Na TPP (Na_5_P_3_O_10_) was dissolved in water to carry hydroxyl and phosphoric ions in the ionic gelation process. This process formed an ionic complex via electrostatic interactions between the amine groups of CS and the trivalent TPP anion [37,38] due to the polycationic nature of CS in acidic solutions (pH 4.0–6.5). Cross-linking enhanced several essential characteristics of the CSNPs, including their stability, mechanical strength, swelling capacity, solubility, and drug release capabilities [39,40,41,42,43,44,45,46].

### 2.3. Characterization of AM-F-CS-NPs.

#### 2.3.1. Physical and Morphology Nanoparticles

Nanoparticle design plays a crucial role in the efficient delivery of drugs due to various biophysical limitations [47]. The AM-F-CS-NP demonstrated the desired diameter, encapsulation efficiency, and spherical shape. Using 20-kDa CS reduced the NP size from 250.9 ± 23 to 180.5 ± 12 nm (Table 2). This smaller size is advantageous, as it favors renal filtration, which has a margin of 3.5 nm, thereby protecting the drug from rapid elimination [48]. Additionally, the smaller size allows for greater penetration into solid tumors with larger vascular cavities (200 to 780 nm) through which the drug-containing NPs can enter [49].

The difference in nanoparticle sizes can be attributed to the type of molecular weight of CS used in the formulation. High molecular weight CS usually contains more amino groups along its long chain, which can result in larger particle sizes when cross-linked to form CSNPs. As a result, more available amino groups allow for more cross-link sites, resulting in larger aggregates or nanoparticles. Second, the molecular weight also represents the length of the CS chain, which can affect the particle-formation process and the resulting nanoparticle structure. Longer CS chains may have a more significant steric effect, with the size and spatial arrangement of the polymer chains affecting the accessibility of the crosslinking sites. This process can lead to more collapsed or denser particles in larger locations due to the limited availability of reactive groups for crosslinking.

Moreover, the ratios of CS, folate, and Na TPP concentrations can influence the surface charge of NPs. The zeta potential of the NPs increased from 10.69 ± 1.3 mV to 40.33 ± 3.4 mV, indicating an increase in their stability. In general, boosting the zeta potential of NPs increases their surface charge, leading to strong repulsive interactions and more excellent stability and uniform size [50]. This improved stability is crucial for effective drug delivery and targeted therapies, making CSNPs a promising strategy in pharmaceutical applications.

In this study, we observed that decreasing the molecular weight of CS significantly reduced the zeta potential of the NPs from 40.33 ± 3.4 mV to 10.69 ± 1.3 mV. CSNPs typically have a positive surface charge due to the protonation of amine groups in acidic pH conditions. When high-molecular weight CS is employed, it can increase the number of positively charged NH_3_^+^ groups on the surface, thereby increasing the surface charge. However, for pH levels other than 4 and 6, neither the type of CS nor its molecular weight significantly impacted the surface charge of the NPs [51,52].

**Figure 2:**
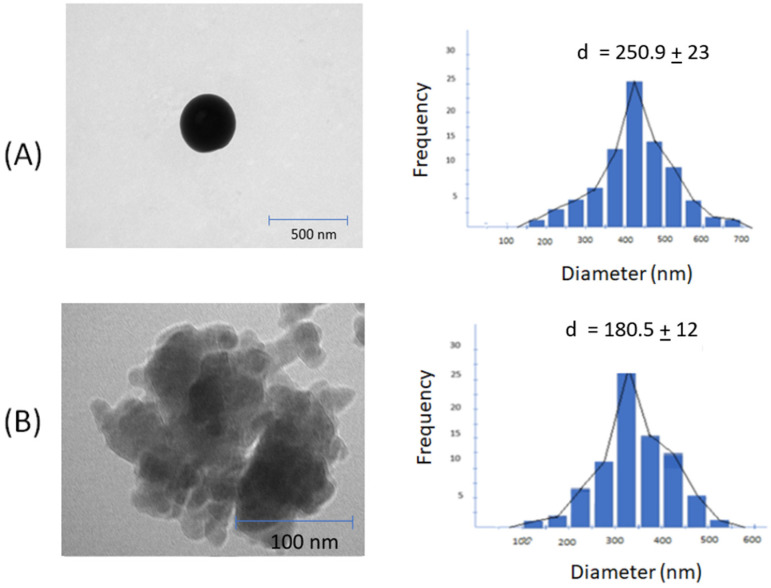
TEM images and size distribution: (**A**) F1 (AM-F-CS-HMW); (**B**) F2 (AM-F-CS-LMW).

Most studies have found that CSNPs exhibit a spherical morphology (Figure 2) [53,54]. The molecular weight of CS does not appear to affect the spherical shape of the particles [55]. Instead, the complexation between oppositely charged species shapes CS into spherical particles [56]. Cancer cells use NPs based on their size, shape, and surface functionalization [57,58].

**Figure 3:**
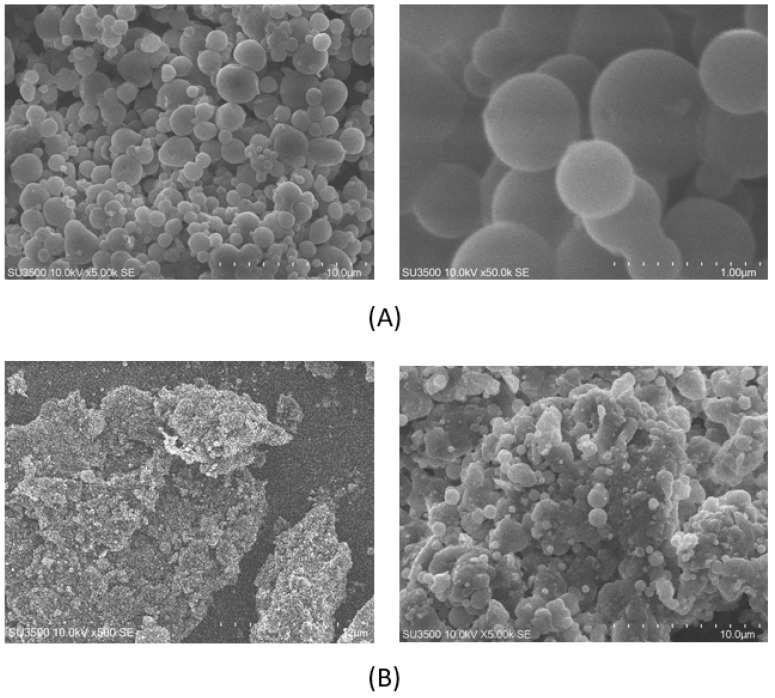
SEM of CSNP surfaces: (**A**) F1 (AM-F-CS-HMW); (**B**) F2 (AM-F-CS-LMW).

The smoothness of nanoparticle surfaces (Figure 3) significantly impacts drug characteristics, particularly in terms of their stability, bioavailability, and targeting ability [59,60]. A smoother surface can enhance the stability of nanoparticles by reducing defects or irregularities that might lead to particle aggregation or degradation. Moreover, a smooth surface facilitates improved drug bioavailability, as it enhances absorption rates and lowers the likelihood of clearance by the immune system. Additionally, smooth nanoparticles are more effective in targeting specific cells or tissues by minimizing non-specific interactions with other cells or molecules [61]. Achieving a smooth nanoparticle surface is crucial for optimizing drug delivery and enhancing therapeutic outcomes [62].

Several factors can influence the smoothness of nanoparticle surfaces, including the synthesis method, surface modification, size and shape, agglomeration, environmental conditions, quality control, surface energy, and composition of the NPs. For instance, in the case of CSNPs, hydrophobicity can affect surface smoothness. Hydrophobic drugs may interact with the hydrophobic regions of CS molecules, leading to aggregate formation and rough surfaces. Conversely, hydrophilic drugs may interact with the hydrophilic regions of CS molecules, resulting in more homogeneous drug distribution and a smoother surface [63]. Furthermore, the synthesis method and surface modification used in preparing CSNPs can also influence the smoothness of the particles. For instance, electrostatic interaction-based methods may result in a more irregular surface due to collision and aggregation. At the same time, ionic gelation may lead to smoother surfaces through cross-linking of the CS [64,65].

Recent studies have shown that the size reduction effect of folate conjugation to CSNPs may depend on various factors, such as the degree of deacetylation of CS and the folate-to-CS ratio [66,67]. A higher degree of deacetylation of CS may result in a more significant reduction in nanoparticle size upon folate conjugation. Additionally, the folate-to-CS ratio can affect the size reduction effect, with a higher folate-to-CS ratio leading to a more substantial decrease in size.

Moreover, the smoothness of CSNPs can affect their drug release, stability, and targeting ability and impact cellular uptake and toxicity. It has been reported that rough surfaces of NPs can induce more cellular uptake and cause higher toxicity due to increased interaction with cell membranes. Therefore, maintaining a smooth surface can reduce toxicity and improve biocompatibility.

In addition, the surface charge of CSNPs can also affect their biological properties. A recent study showed that positively charged CSNPs can enhance cellular uptake and improve therapeutic efficacy compared to negatively charged NPs. However, positively charged NPs may also lead to increased toxicity, which should be carefully evaluated.

Overall, the conjugation of folate to CSNPs can significantly impact their size and biological properties, and these factors should be carefully considered in the design and development of targeted drug-delivery systems [66,68,69].

#### 2.3.2. FTIR Analysis

**Figure 4:**
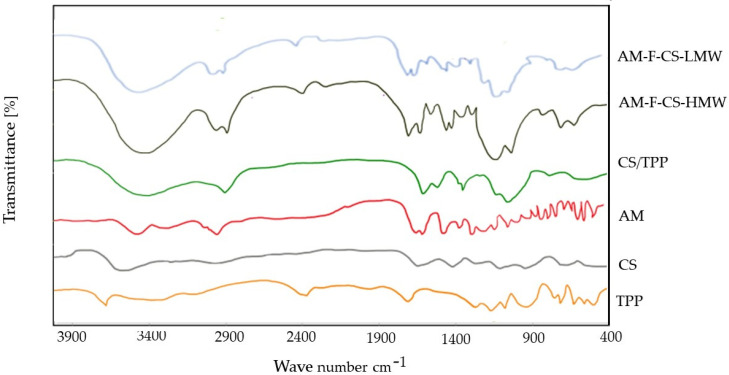
FTIR spectra of AM–F-CS NPs.

Using FTIR to assess AM–CS interactions, the ability of the ionic gelation mechanism to form AM–CS–NPs was evaluated (Figure 4).

Figure 4 displays the FTIR spectrum of CSNPs, where the broad peak in the 3500- to 3300-cm^−1^ region corresponds to hydrogen-bonded O-H stretching vibrations. The primary amine and amide form II N-H peaks overlap, the C-O-C asymmetric stretch peak appears at around 1150 cm^−1^, and the C-N stretch peak of amine type I is observed at 1317 cm^−1^. Notably, in the NPs, the N-H bending vibration of amine I at 1600 cm^−1^ and the carbonyl stretch of amide II at 1650 cm^−1^ shifted to 1540 cm^−1^ and 1630 cm^−1^, respectively, indicating successful nanoparticle formation and functionalization [70,71].

Additionally, the FTIR spectrum of low molecular weight CS (CS-LMW) matched that of the original CS. No band was detected between 1650 and 1900 cm^−1^, indicating the absence of oxidative groups in CS-LMW [72,73].

### 2.4. In Vitro Drug Release

The impact of F-modification on AM release from CSNPs was investigated in vitro, and it was found that there was no significant effect. As shown in Figure 3, the release of the drug is dependent on both pH and time. pH significantly impacts AM-F-CSNP release, as improved protonation of CS amino groups can result in a loose nanoparticle structure and increased AM solubility, which may be responsible for the accelerated drug release at a lower pH. This pH-dependent hydrolysis is advantageous for delivering anticancer medications in the tumor microenvironment, which has a higher acidic pH (6.0–7.0) than normal plasma (7.4) [49].

The in vitro release profile of AM-F-CS-NPs was conducted over 8 h, and the drugs exhibited sustained release behavior, with a gradual increase in total drug release up to 20 h. The formulation displayed continuous release activity. The initial burst release observed can be attributed to the small NPs. As the particle diameter decreased, so did the distance to the drug’s surface, resulting in sustained drug release [34].

**Figure 5:**
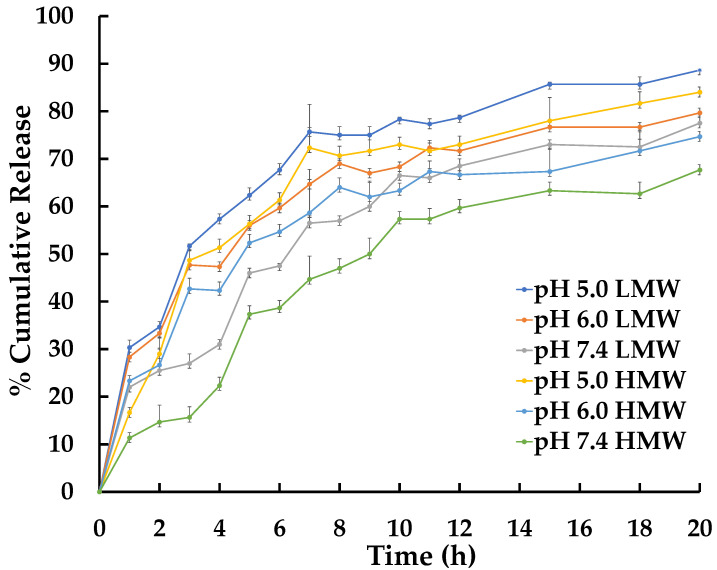
In vitro AM release profiles from AM-F-CS-NPs at 37 °C.

The release data from this study showed that the pH and molecular weight of CS significantly influenced the release of AM from AM-F-NPs (Figure 5). At a low pH, the surface charge of the CSNP becomes positive, reducing electrostatic interactions and facilitating drug release. This pH responsiveness is beneficial under physiological conditions (pH 7.4), in which most of the drug remains within the nanoparticles, causing prolonged circulation and reducing side effects on healthy tissue. However, once drug-containing CSNPs are endocytosed by tumor cells, the lower pH in the tumor microenvironment promotes rapid drug release, potentially increasing the effectiveness of cancer therapy. This pH-dependent drug release mechanism in AM-F-NPs offers a promising approach for targeted and controlled drug delivery in cancer treatment. High molecular CS has a larger number of amino groups, and the steric effect can result in the formation of CSNPs, which are larger and potentially more compact than those formed using CS with a lower molecular weight. Additionally, the particle size of the NPs can impact drug dissolution following the Noyes–Whitney equation. The Noyes–Whitney equation describes the dissolution rate of a solid drug particle and states that the dissolution rate is directly proportional to the surface area of the drug particles [74,75].

It is important to note that drug release and cytotoxicity often have a close relationship. Drug release refers to the process by which drugs are released from the delivery system and become available to the body for therapeutic effects. The rate and duration of drug release can significantly impact the efficacy and safety of a drug. For example, a slow release rate may result in a lower peak drug concentration, reducing toxicity and leading to a lower overall therapeutic effect. Conversely, a drug-delivery system that releases the drug quickly may lead to a higher peak drug concentration and, therefore, higher cytotoxicity. However, a drug-delivery system that releases the drug slowly may lead to a lower peak drug concentration and, hence, lower cytotoxicity and overall therapeutic effect.

Several variables can affect drug release from CSNPs, including the polymer molecular weight, deacetylation, nanoparticle size, porosity, and shape. The ability of drug molecules to interact electrostatically and be incorporated into CS structures depends on their length and conformation [76]. Thus, optimizing these variables can significantly impact the efficacy and safety of drug-delivery systems based on CSNPs.

To understand the kinetic release of AMG from CSNPs within the first 24 h, we conducted an analysis using various mathematical models: zero-order, first-order, Higuchi, and Korsmeyer-Peppas [77]. The correlation coefficients (r) obtained from the CSNP data (shown in Table 3 and Table 4) indicate that there are two types of AMG release from F-CS-NPs:

- At a pH of 5, the release model follows the Higuchi model, suggesting a matrix-type release mechanism based on Fickian diffusion;
- However, at pH values of 6 and 7, the release follows the Korsmeyer–Peppas model, indicating a non-Fickian diffusion process.

The Korsmeyer–Peppas release model determines the value of “n” from a regression equation, with obtained values typically ranging from 0.45 to 1. A value of “n” between 0.45 and 1.0 indicates non-Fickian release, suggesting contributions from mechanisms other than Fickian diffusion. Non-Fickian conditions can lead to faster or slower drug release rates than expected from Fickian diffusion alone. The drug-release mechanism can be further confirmed using Korsmeyer–Peppas plots, with slope values less than 0.5 indicating diffusion-controlled release [78], contributing to more sustained drug release [77]. Analyzing drug release under different pH conditions helps in the design of drug-delivery systems with optimized and controlled release profiles, ultimately enhancing therapeutic outcomes.

### 2.5. In Vitro Cytotoxicity

**Figure 6:**
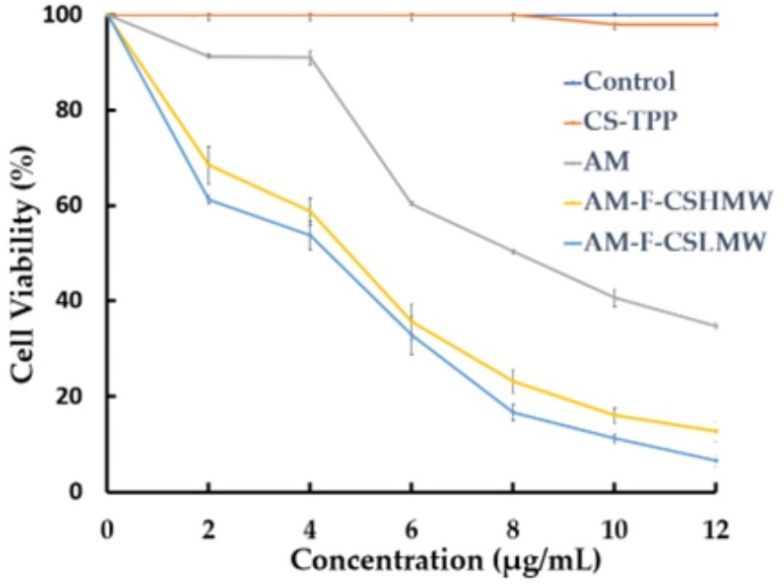
In vitro cytotoxicity of AM-F-CS-NPs.

AM offers anticancer characteristics and is used to treat breast cancer. This research customized CSNPs with folate and AM to target FR-positive cells. No cytotoxic effect on cells was observed for CS-TPP (Figure 6). There was a significant difference between the cytotoxicity of AM and AM-F-CS-NPs. The IC_50_ of AM, AM-F-CS-HMW-NPs, and AM-F-CS-LMW-NPs was 8.47 ± 0.49, 5.3 ± 0.01, and 4.70 ± 0.11 µg/mL, respectively.

**Figure 7:**
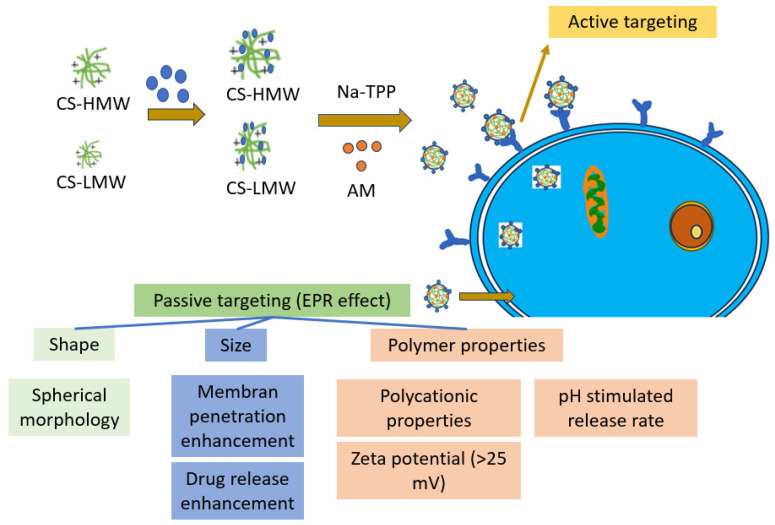
The main aspect of the cytotoxicity enhancement of AM-F-CS-NPs.

The MTT assay is commonly used to assess cytotoxicity. In the current study, the presence of free amine groups and reduced steric hindrance resulting from folate conjugation may have facilitated the growth of CS-NPs. Folate has a strong affinity for folate receptors (FRs) and is stable, cost-effective, and not very immunogenic. F-CS-NPs have been used to encapsulate various anticancer drugs, including doxorubicin, 5-fluorouracil, ursolic acid [79], and cytarabine [46], and as DNA-delivery vehicles [35]. Conjugating polymers with targeting ligands, such as folate, has become a popular approach for targeted drug delivery. The increased internalization of NPs through endocytosis is mostly due to the specific interaction of folate receptors overexpressed in breast cancer cells [69]. In previous studies, passive targeting based on physicochemical properties, such as size and surface charge, has been easily adjustable by adjusting the component molecules or fabrication method [57]. F-CS-AM-NPs may increase AM effectiveness in two ways (Figure 7).

Active targeting through folate-directed AM-F-CS-NPs enhances cytotoxicity, as the MTT results indicate, with more significant inhibition of MCF-7 cancer cell growth and survival compared to AM alone. This capability makes AM-F-CS-NPs a potential solution for overcoming drug resistance in tumors by specifically targeting cells expressing folate receptors, which are often overexpressed in cancer cells [35]. Additionally, AM-F-CS-NPs exhibit high specificity in cellular uptake by folate receptor-expressing cells, offering a suitable vehicle for addressing tumor drug resistance. Regarding passive targeting mechanisms, the shape and size of nanoparticles play crucial roles [80,81]. Spherical polymer NPs of a specific size facilitate membrane penetration and absorption, while the size range of 100–200 nm displayed by AM-F-CS-NPs takes advantage of unique tumor-associated phenomena, such as holes or gaps between endothelial cells in the tumor vasculature and tissue, enabling targeted delivery to cancer cells [82].

Moreover, CS polycationic characteristics allow positively charged AM-F-CS-NPs to be readily absorbed by cancer cells with negatively charged membranes through electrostatic interactions. The zeta potential exceeding 25 mV ensures stable nanoparticle dispersion and absorption by tumor cells. Furthermore, protonation of the CS amino group at a lower pH promotes drug release from the porous matrix of CSNPs, displaying pH adaptive swelling and decreased solubility. These combined active and passive targeting features make AM-F-CS-NPs a promising strategy for overcoming drug resistance in tumors and improving cancer treatment efficacy [83]. Folate conjugation with CS in cancer drug-delivery systems offers significant advantages, such as targeted drug delivery, biocompatibility, and sustained release [32]. However, there are challenges related to synthesis complexity, variable targeting efficiency, and potential immune responses [32,84,85]. Despite these disadvantages, researchers continue to explore and optimize CS-based drug-delivery systems for improved cancer therapy outcomes [86,87].

Molecular modelling and structural aspects are crucial in drug development and delivery [88,89]. They use computer methods to study how drugs interact with target molecules, predict drug behavior, and design delivery systems for better results (targeting, controlled release, stability, biocompatibility, and pharmacokinetics) [90,91]. Using these computational tools, we can discover and optimize new drugs faster, leading to more effective and targeted treatments for different diseases.

## 3. Materials and Methods

### 3.1. Materials

CS (300 kDa) and sodium tripolyphosphate (Na TPP) were purchased from Interlab, Ltd. (Jakarta, Indonesia). Folate (F) and 1-(3-dimethylaminoproply)-3-ethylcarbodiimide hydrochloride (EDC) were purchased from Sigma-Aldrich (St. Louis, MO, USA). MCF-7 cancer cells were provided by the American Type Culture Collection (Manassas, VA, USA).

### 3.2. Preparation of F-CS

Low-molecular weight CS (20 kDa) was prepared as in previous studies [57]. F-CS was synthesized using an aminoacylation process. For the attachment of FA to CS, a 0.2 mol ratio of FA to 1 mol CS was used. Initially, 200 mg of CS (1.24 mmol) was dissolved in 50 mL of 1 M acetic acid. A solution of 100 mg (0.23 mmol) of FA and 50 mg (0.26 mmol) of EDC hydrochloride in 20 mL of DMSO was then added to the CS solution, and the mixture was continuously stirred using a magnetic stirrer for 18 h in the dark. After 18 h, the pH was adjusted to 9 using 1 M sodium hydroxide. CS-modified folic acid was precipitated by centrifuging the mixture at 2500 rpm. F-CS was dissolved in 50 mL of water and dialyzed against PBS for three days, followed by four days against water. The F-CS-containing solution was then freeze-dried and kept at 4 °C.

### 3.3. Characterization F-CS

Fourier-transform infrared spectrophotometer (Model IR Prestige-21, Kyoto, Japan) and a UV-spectrophotometer (Shimadzu UV-1601, Kyoto, Japan) at 363 nm were used to study the CS-chemical F’s structure [35].

### 3.4. Preparation AM-F-CS-NPs

The CSNPs were produced using a modified ionic gelation method, as described in Table 5. Initially, 20 mg of AM was diluted with 20 mL of ethanol and mixed with a 0.1% *w*/*v* (200 mg/200 mL) solution of F-CS in 1% acetic acid. This combination was stirred overnight at a pH of 4.7–4.8. In a separate container, 40 mg of TPP was dissolved in 10 mL of cold (25 °C), filtered (0.22 µm) distilled water [92]. Next, 10 mL of the TPP solution was added to 200 mL of the AM-F-CS solution, using this process to prepare Formula (2) [93].

### 3.5. Characterization of AM-F-CS NPs

#### 3.5.1. Physical Properties and Morphology Nanoparticles

Zetasizer SZ 100 Horiba was used to analyze samples for particle size and zeta potential (Kyoto, Japan) [72]. SEM (Thermo Scientific, Braunschweig, Germany) was used to analyze the NP surface morphology (SEM). Nanoparticle powder was attached on a stub. The powder was conductive with a narrow platinum beam for 30 s at 10 mA. The 10-kV picture is magnified. TEM (Thermo Scientific, Braunschweig, Germany) was utilized to analyze the morphologies of all CSNPs. Before analysis, carbon-coated samples were inspected under a microscope.

#### 3.5.2. Fourier-Transform Infrared Analysis

F-CS was characterized by a Fourier-transform infrared (FTIR) spectrophotometer (Model IR Prestige-21, Kyoto, Japan) at 4000–400 cm^−1^ [70].

### 3.6. Loading Efficiency (LE) and Loading Capacity (LC)

UV-VIS spectroscopy was used to measure the LE of AM and LC of CSNPs. Twenty milligrams of sample NPs were diluted in ethyl acetate before centrifugation (3000 rpm, 10 min). The absorbance of filtrate at 245 nm was determined using UV-visible spectrophotometry to assess the amount of free AM [94]. The total quantity of AM was estimated, and the silt was reconstituted in ethanol to ascertain the LC. The various concentrations (2–20 g/mL) recorded at 245 nm were used to generate a standard curve.

The LE and LC of AM contained in NPs were determined using Equations (1) and (2), respectively [95,96]:

(1) $$\mathrm{Loading}\;\mathrm{Efficiency}\;\left(\%\right)=\frac{\mathrm{mass}\;\mathrm{of}\;\mathrm{AM}\;\mathrm{present}\;\mathrm{in}\;\mathrm{nanoparticle}\;\left(\mathrm{mg}\right)\;}{\mathrm{mass}\;\mathrm{of}\;\mathrm{AM}\;\mathrm{used}\;\left(\mathrm{mg}\right)}\;\times 100\%$$

(2) $$\mathrm{Loading}\;\mathrm{Capacity}\;\left(\%\right)=\frac{\mathrm{mass}\;\mathrm{of}\;\mathrm{AM}\;\mathrm{present}\;\mathrm{in}\;\mathrm{nanoparticle}\;\left(\mathrm{mg}\right)\;}{\mathrm{the}\;\mathrm{total}\;\mathrm{mass}\;\mathrm{of}\;\mathrm{nanoparticle}\;\left(\mathrm{mg}\right)}\;\times 100\%$$

### 3.7. In Vitro Drug Release

To dialyze the AM-F-CS-NPs, 100 mg of the particles were suspended in 50 mL of deionized water for injection and then placed inside a membrane dialysis bag (Ward Science, West Henrietta, NY, USA, MW cut-off 14,000 Da) in PBS at pH values of 5.0, 6.0, and 7.4 with constant stirring at 37 °C. Five milliliters of dialysis buffer was withdrawn from the dialysis bag at regular intervals. Samples were taken periodically for 2, 4, 6, and 8 h, and the AM content was determined by UV-spectrophotometry analysis (Shimadzu UV-1601, Kyoto, Japan) [97,98]. pH 5.0, pH 6.0, and pH 7.4 were used to test AM release from NPs [99,100,101]. We obtained the release profile by plotting the total amount of AM released from the matrix against time in PBS.

The in-vitro drug release data were applied to several kinetic models (zero order, first order, Higuchi’s kinetics, and Korsmeyer’s equation) for formulation in various release media (Table 6). The in-vitro drug release data were fitted to multiple kinetic models to comprehend the drug release and rate-controlling mechanisms for the varied drug release. Every release experiment was conducted in triplicate. For each kinetic model, the drug release mechanism and linearization were calculated by finding the fit quality (R2) and the number of residuals squared (SSR).

### 3.8. In Vitro Cytotoxicity

In a 96-well culture plate, 1 × 10^4^ MCF-7 cells were seeded in 10,000 culture medium and incubated for 24 h. Various quantities of F-CS-AM-NPs (1, 0.5, 0.25, 0.05, or 0.01 mg/mL) were added to the cells and cultured for 48 h at 37 °C. Ten microliters of 0.5% MTT solution was added to each well’s cells, which were then cultured for an additional 4 h before 100 µL of DMSO was added. After dissolving formazan, the plate was agitated. Calculating the metabolism of the tetrazolium substrate, MTT, allowed for the determination of cell growth. The absorbance was at 570 nm. Cell viability declined as absorption dropped. IC_50_ was computed using linear regression’s best-fit line [102]. Similarly, we measured pure AM, CS-TPP, and F-CS-TPP as controls.

### 3.9. Statistical Analysis

The standard deviation of the mean was given, and all measurements were performed in triplicate. The results were analyzed using the *t*-test or Student’s *t*-test. Results were considered statistically significant if their *p*-value was less than or equal to 0.05.

## 4. Conclusions

CSNPs have been the subject of extensive research and are ideal delivery systems for cytotoxic anticancer agents. Compared to conventional NPs, which rely on passivity for tumor targeting and pure AM, the cytotoxicity of CNSPs is increased. F-CS-AM-NP is a potential tool for targeting MCF-7 cells and offers a promising strategy for optimizing therapeutic effectiveness. Further research is needed to clarify how various molecular weights of CS in CSNPs act on other breast cancer cell lines and normal cell lines and their feasibility for clinical application. This study still requires confirmation in vivo and selectivity tests on normal cells.

## Figures and Tables

**Table 1:** UV absorption spectroscopy of CS, F, and F-CS.

Compound	Max Absorbance (λ)	Chromophore Groups
CS	218 nm	N-acetyl glucosamine and glucosamine
F	287 nm	π→π* of the aromatic ring
F-CS	218 nm and 282 nm	The n→π* and π→π* transitions are related to the formation of amide bonds and the transition of C=C bonds, respectively.

**Table 2:** Loading efficiency and loading capacity of the NPs: (A) AM-F-CS-HMW-NPs; (B) AM-F-CS-LMW-NPs.

Formula	Size (nm)	ZP (mV)	Shape	Loading Efficiency (%)	Loading Capacity (%)	PDI
F1	250.9 ± 23	+40.33 ± 3.4	Spherical	88.34 ± 1.30	5.93 ± 0.43	0.513 ± 0.05
F2	180.5 ± 12	+10.69 ± 1.3	Spherical	80.35 ± 1.40	5.43 ± 0.33	0.429 ± 0.03

**Table 3:** Higuchi regression parameter of AM from CSNPs in PBS in 24 h.

Parameter	pH 5.0		pH 6.0		pH 7.4	
	CS–LMW	CS–HMW	CS–LMW	CS–HMW	CS–LMW	CS–HMW
Slope (%h^−0.5^)	28.23 ± 1.48	22.43 ± 2.21	26.77 ± 1.31	19.51 ± 0.42	20.02 ± 2.08	19.10 ± 0.75
Correlation coefficient (r)	0.95 ± 0.02	0.93 ± 0.02	0.92 ± 0.03	0.91 ± 0.01	0.79 ± 0.03	±0.03

**Table 4:** Korsmeyer–Peppas regression parameter of AM from CSNPs in PBS in 24 h.

Parameter	pH 5.0		pH 6.0		pH 7.4	
	CS–LMW	CS–HMW	CS–LMW	CS–HMW	CS–LMW	CS–HMW
Slope (%h^−0.5^)	0.37 ± 0.01	0.361 ± 0.02	0.58 ± 0.01	0.46 ± 0.03	0.51 ± 0.03	0.74 ± 0.03
Correlation coefficient (r)	0.94 ± 0.01	0.92 ± 0.01	0.94 ± 0.03	0.93 ± 0.02	0.92 ± 0.09	0.92 ± 0.01

**Table 5:** AM polymeric nanoparticle formulation.

Formulation	F1	F2
AM (mg)	20	20
F-CS-HMW (mg)	200	
F-CS-LMW (mg)		200
Na TPP (mg)	40	40

**Table 6:** Kinetic models and the equations of kinetic models.

No	Kinetic Models	The Equations of Kinetic Models
1	Zero Order	C = K_o_t
2	First Order	Log C = Log C_o_ − kt/2.303
3	Higuchi’s kinetics	Q = Kt^1/2^
4	Korsmeyer’s equation	Mt/M∞ = Kt^n^

## Data Availability

The data samples are provided by the corresponding author on request.

## References

[1] Iqbal J., Abbasi B.A., Mahmood T., Kanwal S., Ali B., Shah S.A. (2017). Plant-derived anticancer agents: A green anticancer approach. Asian Pac. J. Trop. Biomed..

[2] Shetty V., Jakhade A., Shinde K., Chikate R., Kaul-Ghanekar R. (2021). Folate mediated targeted delivery of cinnamaldehyde loaded and FITC functionalized magnetic nanoparticles in breast cancer: In vitro, in vivo and pharmacokinetic studies. New J. Chem..

[3] Ruman U., Buskaran K., Pastorin G., Masarudin M.J., Fakurazi S., Hussein M.Z. (2021). Synthesis and characterization of chitosan-based nanodelivery systems to enhance the anticancer effect of sorafenib drug in hepatocellular carcinoma and colorectal adenocarcinoma cells. Nanomaterials.

[4] Sartaj A., Qamar Z., Qizilbash F.F., Alhakamy N.A., Baboota S., Ali J. (2021). Polymeric Nanoparticles: Exploring the Current Drug Development and Therapeutic Insight of Breast Cancer Treatment and Recommendations. Polymers.

[5] Angelopoulou A., Kolokithas-Ntoukas A., Fytas C., Avgoustakis K. (2019). Folic Acid-Functionalized, Condensed Magnetic Nanoparticles for Targeted Delivery of Doxorubicin to Tumor Cancer Cells Overexpressing the Folate Receptor. ACS Omega.

[6] Ibrahim M.Y., Hashim N.M., Mariod A.A., Mohan S., Abdulla M.A., Abdelwahab S.I., Arbab I.A. (2016). α-Mangostin from *Garcinia mangostana* Linn: An updated review of its pharmacological properties. Arab. J. Chem..

[7] Setyawati L.U., Nurhidayah W., Khairul Ikram N.K., Mohd Fuad W.E., Muchtaridi M. (2023). General toxicity studies of alpha mangostin from *Garcinia mangostana*: A systematic review. Heliyon.

[8] Rizeq B., Gupta I., Ilesanmi J., Alsafran M., Rahman M. (2020). The Power of Phytochemicals Combination in Cancer Chemoprevention. J. Cancer.

[9] Lee H.N., Jang H.Y., Kim H.J., Shin S.A., Choo G.S., Park Y.S., Kim S.K., Jung J.Y. (2016). Antitumor and apoptosis-inducing effects of α-mangostin extracted from the pericarp of the mangosteen fruit (*Garcinia mangostana* L.) in YD-15 tongue mucoepidermoid carcinoma cells. Int. J. Mol. Med..

[10] Salehi B., Fokou P.V.T., Yamthe L.R.T., Tali B.T., Adetunji C.O., Rahavian A., Mudau F.N., Martorell M., Setzer W.N., Rodrigues C.F. (2019). Phytochemicals in prostate cancer: From bioactive molecules to upcoming therapeutic agents. Nutrients.

[11] Vemu B., Nauman M.C., Veenstra J.P., Johnson J.J. (2019). Structure activity relationship of xanthones for inhibition of Cyclin Dependent Kinase 4 from mangosteen (*Garcinia mangostana* L.). Int. J. Nutr..

[12] Novilla A., Djamhuri D.S., Fauziah N., Maesaroh M., Balqis B., Widowati W. (2016). Cytotoxic Activity of Mangosteen (*Garcinia mangostana* L.) Peel Extract and α-Mangostin toward Leukemia Cell Lines (HL-60 and K-562). J. Nat. Remedies.

[13] Li I., Han A., Hamburger M., Kinghorn D., Butterweck V.L.B. (2010). Determination of alpha-mangostin in rat plasma by HPLC-MS and its application to pharmacokinetic studies. Planta Med..

[14] Zhao Y., Tang G., Tang Q., Zhang J., Hou Y., Cai E., Liu S., Lei D., Zhang L., Wang S. (2016). A Method of Effectively Improved α-Mangostin Bioavailability. Eur. J. Drug Metab. Pharmacokinet..

[15] Kondo M., Zhang L., Ji H., Kou Y., Ou B. (2009). Bioavailability and antioxidant effects of a xanthone-rich mangosteen (*Garcinia mangostana*) product in humans. J. Agric. Food Chem..

[16] Yao L., Gu X., Song Q., Wang X., Huang M., Hu M., Hou L., Kang T., Chen J., Chen H. (2016). Nanoformulated alpha-mangostin ameliorates Alzheimer’s disease neuropathology by elevating {LDLR} expression and accelerating amyloid-beta clearance. J. Control. Release.

[17] Herdiana Y., Wathoni N., Shamsuddin S., Muchtaridi M. (2021). α-Mangostin nanoparticles cytotoxicity and cell death modalities in breast cancer cell lines. Molecules.

[18] Wathoni N., Rusdin A., Motoyama K., Joni I.M., Lesmana R., Muchtaridi M. (2020). Nanoparticle drug delivery systems for α-mangostin. Nanotechnol. Sci. Appl..

[19] Sakpakdeejaroen I., Muanrit P., Panthong S., Ruangnoo S. (2022). Alpha-Mangostin-Loaded Transferrin-Conjugated Lipid-Polymer Hybrid Nanoparticles: Development and Characterization for Tumor-Targeted Delivery. Sci. World J..

[20] Verma R.K., Yu W., Shrivastava A., Shankar S., Srivastava R.K. (2016). α-Mangostin-encapsulated PLGA nanoparticles inhibit pancreatic carcinogenesis by targeting cancer stem cells in human, and transgenic (KrasG12D, and KrasG12D/tp53R270H) mice. Sci. Rep..

[21] Wathoni N., Rusdin A., Febriani E., Purnama D., Daulay W., Azhary S.Y., Panatarani C., Joni I.M., Lesmana R., Motoyama K. (2019). Formulation and Characterization of α-Mangostin in Chitosan Nanoparticles Coated by Sodium Alginate, Sodium Silicate, and Polyethylene Glycol. J. Pharm. Bioallied Sci..

[22] Wathoni N., Meylina L., Rusdin A., Mohammed A.F.A., Tirtamie D., Herdiana Y., Motoyama K., Panatarani C., Joni I.M., Lesmana R. (2021). The Potential Cytotoxic Activity Enhancement of α-Mangostin in Chitosan-Kappa Carrageenan-Loaded Nanoparticle against MCF-7 Cell Line. Polymers.

[23] Meylina L., Muchtaridi M., Joni I.M., Elamin K.M., Wathoni N. (2023). Hyaluronic Acid-Coated Chitosan Nanoparticles as an Active Targeted Carrier of Alpha Mangostin for Breast Cancer Cells. Polymers.

[24] Samprasit W., Opanasopit P. (2020). Chitosan-Based Nanoparticles for Controlled-Release Delivery of α–Mangostin. Int. J. Pharma Med. Biol. Sci..

[25] Gatoo M.A., Naseem S., Arfat M.Y., Dar A.M., Qasim K., Zubair S. (2014). Physicochemical Properties of Nanomaterials: Implication in Associated Toxic Manifestations. Biomed Res. Int..

[26] Khan I., Saeed K., Khan I. (2019). Nanoparticles: Properties, applications and toxicities. Arab. J. Chem..

[27] Hoosain F.G., Choonara Y.E., Tomar L.K., Kumar P., Tyagi C., du Toit L.C., Pillay V. (2015). Bypassing P-Glycoprotein Drug Efflux Mechanisms: Possible Applications in Pharmacoresistant Schizophrenia Therapy. Biomed Res. Int..

[28] Yanat M., Schroën K. (2021). Preparation methods and applications of chitosan nanoparticles; with an outlook toward reinforcement of biodegradable packaging. React. Funct. Polym..

[29] Moraru C., Mincea M., Menghiu G., Ostafe V. (2020). Understanding the Factors Influencing Chitosan-Based Nanoparticles-Protein Corona Interaction and Drug Delivery Applications. Molecules.

[30] Herdiana Y., Wathoni N., Shamsuddin S., Joni I.M., Muchtaridi M. (2021). Chitosan-Based Nanoparticles of Targeted Drug Delivery System in Breast Cancer Treatment. Polymers.

[31] Jafernik K., Ładniak A., Blicharska E., Czarnek K., Ekiert H., Wiącek A.E., Szopa A. (2023). Chitosan-Based Nanoparticles as Effective Drug Delivery Systems—A review. Molecules.

[32] Farran B., Montenegro R.C., Kasa P., Pavitra E., Huh Y.S., Han Y.-K., Kamal M.A., Nagaraju G.P., Rama Raju G.S. (2020). Folate-conjugated nanovehicles: Strategies for cancer therapy. Mater. Sci. Eng. C.

[33] Pawar A., Singh S., Rajalakshmi S., Shaikh K., Bothiraja C. (2018). Development of fisetin-loaded folate functionalized pluronic micelles for breast cancer targeting. Artif. Cells, Nanomed. Biotechnol..

[34] Ortiz-Islas E., Sosa-Arróniz A., Manríquez-Ramírez M.E., Rodríguez-Pérez C.E., Tzompantzi F., Padilla J.M. (2021). Mesoporous silica nanoparticles functionalized with folic acid for targeted release Cis-Pt to glioblastoma cells. Rev. Adv. Mater. Sci..

[35] Cheng L., Ma H., Shao M., Fan Q., Lv H., Peng J., Hao T., Li D., Zhao C., Zong X. (2017). Synthesis of folate-chitosan nanoparticles loaded with ligustrazine to target folate receptor positive cancer cells. Mol. Med. Rep..

[36] Baibarac M., Smaranda I., Nila A., Serbschi C. (2019). Optical properties of folic acid in phosphate buffer solutions: The influence of pH and UV irradiation on the UV-VIS absorption spectra and photoluminescence. Sci. Rep..

[37] Popova E.V., Zorin I.M., Domnina N.S., Novikova I.I., Krasnobaeva I.L. (2020). Chitosan—Tripolyphosphate Nanoparticles: Synthesis by the Ionic Gelation Method, Properties, and Biological Activity. Russ. J. Gen. Chem..

[38] Bhumkar R.D., Pokharkar V.B. (2006). Studies on effect of pH on cross-linking of Chitosan with sodium tripolyphosphate: A technical note. AAPS PharmSciTech.

[39] Jóźwiak T., Filipkowska U., Szymczyk P., Rodziewicz J., Mielcarek A. (2017). Effect of ionic and covalent crosslinking agents on properties of chitosan beads and sorption effectiveness of Reactive Black 5 dye. React. Funct. Polym..

[40] Westlake J.R., Laabei M., Jiang Y., Yew W.C., Smith D.L., Burrows A.D., Xie M. (2023). Vanillin Cross-Linked Chitosan Film with Controlled Release of Green Tea Polyphenols for Active Food Packaging. ACS Food Sci. Technol..

[41] Sharmin N., Rosnes J.T., Prabhu L., Böcker U., Sivertsvik M. (2022). Effect of Citric Acid Cross Linking on the Mechanical, Rheological and Barrier Properties of Chitosan. Molecules.

[42] Han X., Wen H., Luo Y., Yang J., Xiao W., Xie J. (2022). Effects of chitosan modification, cross-linking, and oxidation on the structure, thermal stability, and adsorption properties of porous maize starch. Food Hydrocoll..

[43] Mathew S.A., Arumainathan S. (2022). Crosslinked Chitosan-Gelatin Biocompatible Nanocomposite as a Neuro Drug Carrier. ACS Omega.

[44] Dey S., Majumdar S., Hasnain M.S., Nayak A.K., Hasnain M.S., Beg S., Nayak A.K.B.T.-C. (2022). Chapter 11—Cross-linking of chitosan in drug delivery. Chitosan in Drug Delivery.

[45] Tariq H., Rehman A., Kishwar F., Raza Z.A. (2022). Citric Acid Cross-Linking of Chitosan Encapsulated Spearmint Oil for Antibacterial Cellulosic Fabric. Polym. Sci. Ser. A.

[46] Geethakumari D., Bhaskaran Sathyabhama A., Raji Sathyan K., Mohandas D., Somasekharan J.V., Thavarool Puthiyedathu S. (2022). Folate functionalized chitosan nanoparticles as targeted delivery systems for improved anticancer efficiency of cytarabine in MCF-7 human breast cancer cell lines. Int. J. Biol. Macromol..

[47] Ye H., Shen Z., Yu L., Wei M., Li Y. (2018). Manipulating nanoparticle transport within blood flow through external forces: An exemplar of mechanics in nanomedicine. Proc. R. Soc. A Math. Phys. Eng. Sci..

[48] Claveau S., Nehlig É., Garcia-Argote S., Feuillastre S., Pieters G., Girard H.A., Arnault J.-C., Treussart F., Bertrand J.-R. (2020). Delivery of siRNA to Ewing Sarcoma Tumor Xenografted on Mice, Using Hydrogenated Detonation Nanodiamonds: Treatment Efficacy and Tissue Distribution. Nanomaterials.

[49] Wang Z., Deng X., Ding J., Zhou W., Zheng X., Tang G. (2018). Mechanisms of drug release in pH-sensitive micelles for tumour targeted drug delivery system: A review. Int. J. Pharm..

[50] Nokhodi F., Nekoei M., Goodarzi M.T. (2022). Hyaluronic acid-coated chitosan nanoparticles as targeted-carrier of tamoxifen against MCF7 and TMX-resistant MCF7 cells. J. Mater. Sci. Mater. Med..

[51] Katas H., Hussain Z., Ling T.C. (2012). Chitosan Nanoparticles as a Percutaneous Drug Delivery System for Hydrocortisone. J. Nanomater..

[52] Bruinsmann F.A., Pigana S., Aguirre T., Souto G.D., Pereira G.G., Bianchera A., Fasiolo L.T., Colombo G. (2019). Chitosan-Coated Nanoparticles: Effect of Chitosan Molecular Weight on Nasal Transmucosal Delivery. Pharmaceutics.

[53] Agarwal M., Agarwal M.K., Shrivastav N., Pandey S., Das R., Gaur P. (2018). Preparation of Chitosan Nanoparticles and their In-vitro Characterization. Int. J. Life. Sci. Sci. Res..

[54] Hembram K.C., Prabha S., Chandra R., Nimesh S., Hembram K.C., Prabha S., Chandra R., Ahmed B., Nimesh S. (2016). Advances in preparation and characterization of chitosan nanoparticles for therapeutics. Artif. Cells Nanomed. Biotechnol..

[55] Sreekumar S., Goycoolea F.M., Moerschbacher B.M., Rivera-Rodriguez G.R. (2018). Parameters influencing the size of chitosan-TPP nano- and microparticles. Sci. Rep..

[56] Giri T.K. (2016). Nanoarchitectured Polysaccharide-Based Drug Carrier for Ocular Therapeutics.

[57] Herdiana Y., Wathoni N., Shamsuddin S., Muchtaridi M. (2022). Cytotoxicity Enhancement in MCF-7 Breast Cancer Cells with Depolymerized Chitosan Delivery of α-Mangostin. Polymers.

[58] Dasgupta S., Auth T., Gompper G. (2014). Shape and Orientation Matter for the Cellular Uptake of Nonspherical Particles. Nano Lett..

[59] Ghorbani F., Kokhaei P., Ghorbani M., Eslami M. (2020). Application of different nanoparticles in the diagnosis of colorectal cancer. Gene Rep..

[60] Yaqoob A.A., Sekeri S.H., Othman M.B.H., Ibrahim M.N.M., Feizi Z.H. (2021). Thermal degradation and kinetics stability studies of oil palm (*Elaeis Guineensis*) biomass-derived lignin nanoparticle and its application as an emulsifying agent. Arab. J. Chem..

[61] Bierhalz A.C.K., Westin C.B., Moraes Â.M. (2016). Comparison of the properties of membranes produced with alginate and chitosan from mushroom and from shrimp. Int. J. Biol. Macromol..

[62] Liu F., Xu J., Wu L., Zheng T., Han Q., Liang Y., Zhang L., Li G., Yang Y. (2021). The Influence of the Surface Topographical Cues of Biomaterials on Nerve Cells in Peripheral Nerve Regeneration: A Review. Stem Cells Int..

[63] George D., Maheswari P.U., Begum K.M.M.S. (2020). Chitosan-cellulose hydrogel conjugated with L-histidine and zinc oxide nanoparticles for sustained drug delivery: Kinetics and in-vitro biological studies. Carbohydr. Polym..

[64] Richardson J.J., Björnmalm M., Caruso F. (2015). Technology-driven layer-by-layer assembly of nanofilms. Science.

[65] Ahmed M.E., Saber D., Abd Elaziz K., Alghtani A.H., Felemban B.F., Ali H.T., Megahed M. (2021). Chitosan-based nanocomposites: Preparation and characterization for food packing industry. Mater. Res. Express.

[66] Ullah S., Azad A.K., Nawaz A., Shah K.U., Iqbal M., Albadrani G.M., Al-Joufi F.A., Sayed A.A., Abdel-Daim M.M. (2022). 5-Fluorouracil-Loaded Folic-Acid-Fabricated Chitosan Nanoparticles for Site-Targeted Drug Delivery Cargo. Polymers.

[67] Radnia F., Mohajeri N., Hashemi F., Imani M., Zarghami N. (2021). Design and development of folate-chitosan/CD nanogel: An efficient fluorescent platform for Cancer-specific delivery of AntimiR-21. React. Funct. Polym..

[68] San H.H.M., Alcantara K.P., Bulatao B.P.I., Sorasitthiyanukarn F.N., Nalinratana N., Suksamrarn A., Vajragupta O., Rojsitthisak P., Rojsitthisak P. (2023). Folic Acid-Grafted Chitosan-Alginate Nanocapsules as Effective Targeted Nanocarriers for Delivery of Turmeric Oil for Breast Cancer Therapy. Pharmaceutics.

[69] Ruman U., Buskaran K., Bullo S., Pastorin G., Masarudin M.J., Fakurazi S., Hussein M.Z. (2021). Sorafenib and 5-Fluorouracil Loaded Dual Drug Nanodelivery Systems for Hepatocellular Carcinoma and Colorectal Adenocarcinoma. Res. Sq..

[70] Herdiana Y., Handaresta D.F., Joni I.M., Wathoni N., Muchtaridi M. (2020). Synthesis of nano-α mangostin based on chitosan and Eudragit S 100. J. Adv. Pharm. Technol. Res..

[71] Saraf N.S. (2020). Formulation and evaluation of antifungal agent in a hydrogel containing nanoparticle of low molecular weight chitosan. Int. J. Res. Pharm. Sci..

[72] Zheng X., Yin Y., Jiang W., Xing L., Pu J. (2015). Synthesis and Characterization of Low Molecular Weight Chitosan. BioResources.

[73] Ma Z., Wang W., Wu Y., He Y., Wu T. (2014). Oxidative Degradation of Chitosan to the Low Molecular Water-Soluble Chitosan over Peroxotungstate as Chemical Scissors. PLoS ONE.

[74] Yang H.C., Hon M.H. (2010). The effect of the degree of deacetylation of chitosan nanoparticles and its characterization and encapsulation efficiency on drug delivery. Polym. Plast. Technol. Eng..

[75] do Nascimento E.G., de Caland L.B., de Medeiros A.S.A., Fernandes-Pedrosa M.F., Soares-Sobrinho J.L., dos Santos K.S.C.R., da Silva-Júnior A.A. (2017). Tailoring drug release properties by gradual changes in the particle engineering of polysaccharide chitosan based powders. Polymers.

[76] Lucio D., Martínez-Ohárriz M.C. (2017). Chitosan: Strategies to Increase and Modulate Drug Release Rate. Biol. Act. Appl. Mar. Polysacch..

[77] Fernández-Romero A.M., Maestrelli F., Mura P.A., Rabasco A.M., González-Rodríguez M.L. (2018). Novel findings about double-loaded curcumin-in-HPβcyclodextrin-in liposomes: Effects on the lipid bilayer and drug release. Pharmaceutics.

[78] Basak S., Kumar K., Ramlingam M. (2008). Design and release characteristics of sustained release tablet containing metformin HCl. Brazilian J. Pharm. Sci..

[79] Jin H., Pi J., Yang F., Jiang J., Wang X., Bai H., Shao M., Huang L., Zhu H., Yang P. (2016). Folate-Chitosan Nanoparticles Loaded with Ursolic Acid Confer Anti-Breast Cancer Activities in vitro and in vivo. Sci. Rep..

[80] Augustine R., Hasan A., Primavera R., Wilson R.J., Thakor A.S., Kevadiya B.D. (2020). Cellular uptake and retention of nanoparticles: Insights on particle properties and interaction with cellular components. Mater. Today Commun..

[81] Hatem S., Elkheshen S.A., Kamel A.O., Nasr M., Moftah N.H., Ragai M.H., Elezaby R.S., El Hoffy N.M. (2022). Functionalized chitosan nanoparticles for cutaneous delivery of a skin whitening agent: An approach to clinically augment the therapeutic efficacy for melasma treatment. Drug Deliv..

[82] Herdiana Y., Wathoni N., Gozali D., Shamsuddin S., Muchtaridi M. (2023). Chitosan-Based Nano-Smart Drug Delivery System in Breast Cancer Therapy. Pharmaceutics.

[83] Almualla M.A., Mousa M.N., Sattar M. (2021). Chemical modification and characterization of chitosan for pharmaceutical applications. Egypt. J. Chem..

[84] Yusuf A., Almotairy A.R.Z., Henidi H., Alshehri O.Y., Aldughaim M.S. (2023). Nanoparticles as Drug Delivery Systems: A Review of the Implication of Nanoparticles’ Physicochemical Properties on Responses in Biological Systems. Polymers.

[85] Mitchell M.J., Billingsley M.M., Haley R.M., Wechsler M.E., Peppas N.A., Langer R. (2021). Engineering precision nanoparticles for drug delivery. Nat. Rev. Drug Discov..

[86] Li X., Gao Y., Li H., Majoral J.-P., Shi X., Pich A. (2023). Smart and bioinspired systems for overcoming biological barriers and enhancing disease theranostics. Prog. Mater. Sci..

[87] Lu Y., Luo Q., Jia X., Tam J.P., Yang H., Shen Y., Li X. (2023). Multidisciplinary strategies to enhance therapeutic effects of flavonoids from Epimedii Folium: Integration of herbal medicine, enzyme engineering, and nanotechnology. J. Pharm. Anal..

[88] Bunker A., Róg T. (2020). Mechanistic Understanding from Molecular Dynamics Simulation in Pharmaceutical Research 1: Drug Delivery. Front. Mol. Biosci..

[89] Adelusi T.I., Oyedele A.-Q.K., Boyenle I.D., Ogunlana A.T., Adeyemi R.O., Ukachi C.D., Idris M.O., Olaoba O.T., Adedotun I.O., Kolawole O.E. (2022). Molecular modeling in drug discovery. Inform. Med. Unlocked.

[90] Katiyar R.S., Jha P.K. (2018). Molecular simulations in drug delivery: Opportunities and challenges. WIREs Comput. Mol. Sci..

[91] Ferreira L.G., Dos Santos R.N., Oliva G., Andricopulo A.D. (2015). Molecular Docking and Structure-Based Drug Design Strategies.

[92] Srikulkit K. (2011). Preparation of Depolymerized Chitosan and Its Effect on Dyeability of Preparation of Depolymerized Chitosan and Its Effect on Dyeability of Mangosteen Dye. Chiang Mai J. Sci..

[93] Al-Nerawi N.K., Alsharif S.S.M., Dave R.H. (2018). Preparation of Chitosan-TPP Nanoparticles: The Influence of Chitosan Polymeric Properties and Formulation Variables. Int. J. Appl. Pharm..

[94] Asasutjarit R., Meesomboon T., Adulheem P. (2019). Physicochemical properties of alpha-mangostin loaded nanomeulsions prepared by ultrasonication technique. Heliyon.

[95] Garms B.C., Poli H., Baggley D., Han F.Y., Whittaker A.K., Anitha A., Grøndahl L. (2021). Evaluating the effect of synthesis, isolation, and characterisation variables on reported particle size and dispersity of drug loaded PLGA nanoparticles. Mater. Adv..

[96] Lee K.H., Khan F.N., Cosby L., Yang G., Winter J.O. (2021). Polymer Concentration Maximizes Encapsulation Efficiency in Electrohydrodynamic Mixing Nanoprecipitation. Front. Nanotechnol..

[97] Mulia K., Rachman D., Krisanti E.A. (2019). Preparation, characterization and release profile of chitosan alginate freeze dried matrices loaded with mangostins Preparation, characterization and release profile of chitosan alginate freeze dried matrices loaded with mangostins. J. Phys..

[98] Aisha A.F.A., Abu-Salah K.M., Ismail Z., Shah A.M., Majid A. (2013). Determination of total xanthones in *Garcinia mangostana* fruit rind extracts by ultraviolet (UV) spectrophotometry. J. Med. Plants Res..

[99] Cao M., Luo X., Wu K., He X. (2021). Targeting lysosomes in human disease: From basic research to clinical applications. Signal Transduct. Target. Ther..

[100] Lee S., Shanti A. (2021). Effect of exogenous ph on cell growth of breast cancer cells. Int. J. Mol. Sci..

[101] Pérez-Herrero E., Fernández-Medarde A. (2021). The reversed intra- and extracellular pH in tumors as a unified strategy to chemotherapeutic delivery using targeted nanocarriers. Acta Pharm. Sin. B.

[102] Abolhasani M.H., Safavi M., Goodarzi M.T., Kassaee S.M., Azin M. (2018). Identification and anti-cancer activity in 2D and 3D cell culture evaluation of an Iranian isolated marine microalgae. DARU J. Pharm. Sci..

